# Reproductive toxicity potentials of methanolic extract of *Portulaca oleracea* in male rats: An experimental study

**DOI:** 10.18502/ijrm.v19i3.8572

**Published:** 2021-03-21

**Authors:** Izuchukwu Azuka Okafor, Uchenna Somtochukwu Nnamah, Selasie Ahiatrogah, Dorcas Serwaa, Jude Nnaka

**Affiliations:** ^1^Department of Anatomy, Faculty of Basic Medical Sciences, College of Health Sciences, Nnamdi Azikiwe University, Nnewi Campus, Nnewi, Nigeria.; ^2^Department of Obstetrics and Gynecology, College of Medicine, University of Ibadan, Ibadan, Nigeria.; ^3^Pan African University of Life and Earth Science Institute (Including Health and Agriculture), PAULESI, University of Ibadan, Ibadan, Nigeria.; ^4^Hematology Department, Babcock University Teaching Hospital, Ilisan Remo, Ogun State, Nigeria.

**Keywords:** Portulaca oleracea, Purslane, Testis, Epididymis, Rat, Sperm motility.

## Abstract

**Background:**

Purslane is an edible widely distributed shrub and one of the herbs used in decoctions for the treatment of different ailments including infertility. However, there is a shortage of evidence to validate its reproductive effects.

**Objective:**

To investigate the effect of methanolic extract of *Portulaca oleracea *(MEPO) on the reproductive system of male rats.

**Materials and Methods:**

Fifteen 10-wk old male Wistar rats with an average weight of 183 gr were randomly divided into three groups (n = 5/each). Group A (the control group) received distilled water only; group B received 400 mg/kg MEPO; and group C received 800 mg/kg MEPO for 14 days. The animals fasted overnight after the 14${}^{\mathrm{th}}$ day of administration and euthanized by cervical dislocation. Blood samples, sperm, testes, and epididymis were collected for serum hormones, sperm, and histological analyses.

**Results:**

There was no significant change in the serum luteinizing hormone and testosterone levels across all groups when compared to the control. However, group C showed a significant increase (p = 0.020) in follicle-stimulating hormone levels when compared to the control. There was a significant reduction (p = 0.006) in the sperm count in group C when compared with the control group. There was also a significantly reduced (p = 0.003) sperm motility in MEPO-treated groups compared to the control. While the testis showed no abnormalities in its histoarchitecture across groups, the epididymis showed some blood congestion in MEPO-treated groups.

**Conclusion:**

*Portulaca oleracea *showed the ability to reduce sperm count and motility at higher doses.

## 1. Introduction

The information available over the past five years revealed that in one-third of the cases of infertility, significant pathology is found in the male partners, and in 20% of the cases, in both partners. Males are solely responsible for about 20-30% of infertility cases and contribute to 40-50% of cases overall (1). The testicles, the primary male reproductive organ, fulfill two key functions: the production of male gamete (spermatozoa) and the secretion of hormones, particularly the male hormone, testosterone (TT) (2, 3). The importance of this singular function in males is sacrosanct and forms a major part of overall healthcare except in cases of willful contraception. Production of sperms from the stem cells of the testes is a complex process that requires about 64 days in humans (4).

Sertoli cells play a key function in spermatogenesis by the nourishment of developing sperm cells and also acting as phagocytes consuming residual cytoplasm during spermatogenesis (2), whereas the Leydig cells are solely responsible for androgen production (3). Alteration in the functions of these cells may lead to changes in the hormonal balance and impaired male fertility.

An estimated 80% or more of the world's population depends primarily on herbs for their healthcare. This reliance in medicine derived from local plants is especially prime in developing countries where contemporary Western medicine is often inaccessible or is simply too costly (5). The use of herbs in the management of different ailments is widely practiced, and also it is known that herbs or medicinal plants are plants having natural active ingredients used to remedy diseases and also get rid of pain (6, 7). The important values of some plants have long been published; however, a number of them remain untapped yet, therefore there is a necessity to discover their effects on the human body.

The name *Portulaca* is derived from the Latin word “Porto” meaning “to carry” and “lac” meaning “milk” since the plant has a milky juice (7); while *oleracea* has been derived from Latin, meaning “pertaining to kitchen gardens”, denoting its use as a vegetable. The use of this plant as a vegetable, spice, and medicine has been known since the times of the ancient Egyptians and was prevalent in England during the middle ages (8). It is used homeopathically in Ghana for heart-palpitations (8). The plant is used as a diuretic in Nigeria (9). A tea of the plant is taken in Trinidad as a vermifuge (10). In some areas near Benin City (Nigeria), the plant, along with other constituents is taken to enhance reproductive functions for males and the development of the fetus in females (9). It has been reported that aqueous and methanolic extracts of *Portulaca oleracea* (*MEPO*) have contractile effects, protective effects on hypoxic nerve tissue, skeletal muscle relaxant, and nutritive and antioxidant properties (11). *Portulaca oleracea* (*PO*) is also reported to increase the antioxidant enzyme activities in hepatic, renal, and testicular tissues (12, 13).

This study evaluated the reproductive effects of MEPO in adult Wistar rats.

## 2. Materials and Methods

### Plant collection, identification, and extraction

The fresh aerial parts including stems and leaves of PO was collected from the gardens of Botany Department, Nnamdi Azikiwe University, and authenticated at the Department of Botany, Ambrose Ali University, with herbarium reference number 155A. The plant was washed, cut into smaller parts (for easy drying), shade-dried for two weeks, and finely powdered with a mechanical grinder. The powdered plant sample was soaked in a stoppered container with methanol for three days (72 hr) with frequent agitation. After the three days, the mixture was passed, filtered, and concentrated using a water bath at a temperature of 40°C (7); the yield of the extract was weighed and recorded as 65.75 gr.

### Animal care and handling

Fifteen 10-wk old male Wistar rats with an average weight of 183 gr were obtained from the College of Health Sciences' Animal House, Nnamdi Azikiwe University between September 2018 and January 2019 and acclimatized for 2 wk to eliminate any intercurrent infection under normal housing condition (ventilated room with 12-hr light/dark cycle at 24 ± 2°C) in the animal house of the Anatomy Department, Nnamdi Azikiwe University. The rats were served with standard rat chow and water during the course of the experiment. The Wistar rats were acclimatized for two (2) wk after which the extracts were administered for 14 days.

### Experimental procedure

This is an experimental study. The resource equation for sample size calculation for animal studies was used to determine the sample size for this study (14). Male Wistar rats were randomly divided into three groups with five animals each. A solution of 1 gr/ 20 ml distilled water was constituted with MEPO on each day of administration. The needed concentration for each animal was determined and taken from this stock and the remnant of the constituted extract was discarded after each day of administration.

Group A served as the control group and received only food and distilled water. Group B received 400 mg/kg of MEPO per animal body weight, while group C received 800 mg/kg of MEPO per animal body weight. The same doses were given for 14 days. The water consumption and feeding pattern of the rats before and after the administration periods were observed along with the physical behavioral changes in response to the administration. The extract administration doses were chosen based on previous studies (12, 13).

### Animal sacrifice and sample collection

The animals fasted overnight after the last dosing on the 14${}^{\mathrm{th}}$ day of extract administration. On the 15${}^{\mathrm{th}}$ day, the final body weight was taken and the animals were anesthetized using chloroform. Blood samples were collected by orbital puncture and put in plain tubes with appropriate labels. The tubes were kept standing for 5 min before centrifugation, and the sera were separated and stored at -20°C for further analyses.

The animals were sacrificed to harvest the tissues of interest. The right and left epididymis were excised and weighed. Sperm cells were collected from the left epididymis by crushing it on a petri dish and used immediately for both quantitative and qualitative sperm analysis. The testes were harvested, weighed, and rinsed for each animal. Both the testis and epididymis were transferred to coded containers with freshly prepared Bouin's fluid for further tissue processing.

### Epididymal sperm assessment

To access the progressive sperm motility, the semen was squeezed from the caudal left epididymis into a glass slide and examined under the microscope for sperm motility. The percentage of motile sperms was defined as the number of motile sperms divided by the total number of counted sperms. The sperm viability and morphology were evaluated by adding two drops of warm Eosin-Nigrosin stain to the semen; the sample was uniformly smeared and air-dried and examined for live sperm cells, type, and a number of abnormal spermatozoa. The sperm density was assessed using the left caudal epididymis. The epididymal fluid ratio of 1:20 was prepared by adding 0.1 ml of fluid to 1.9 ml of water, mixed, and the sperm were counted in a Neubauer's counting chamber. The epididymal sperm analysis protocol described in the manual of basic semen analysis was used in this study (15).

### Histopathology 

Tissues were fixed in Bouin's fluid, passed through ascending grades of alcohol, and then through xylene, and embedded in paraffin. Tissues were sectioned at 5 µm and stained with hematoxylin and eosin (H&E) to give contrasting colors to different elements of the cells or tissue thus making them conspicuous and easy to study. Tissue processing protocol used for light microscopy was as described by Geoffrey Rolls (16).

### Hormonal assay

Blood serum samples were analyzed for follicle-stimulating hormone (FSH), luteinizing hormone (LH), and TT levels using the AccuBind enzyme-linked immunosorbent assay (ELISA) microwells for FSH, LH, and TT, purchased from Monobind Inc. Lake Forest, California, USA (17).

### Ethical considerations

This experimental study was approved by the Animal Research Ethics Committee of the Department of Anatomy, Faculty of Basic Medical Sciences, College of Health Sciences, Nnamdi Azikiwe University, Nnewi Campus, Nigeria. The experimental procedures complied with ARRIVE guidelines (18) and the National Institutes of Health Guide for the Care and Use of Laboratory Animals (19).

### Statistical analysis

The results were expressed as mean ± standard error of the mean (SEM) and all values were considered significant at p < 0.05. The data for TT, FSH, LH, and relative organ weight were analyzed using a one-way analysis of variance (ANOVA), followed by a post-hoc test using the least significant difference (LSD). The body weight was analyzed using student dependent *t* test.

## 3. Results

### Body weight measurement

While a significant increase (p = 0.041) was observed in the body weight in the control group and group treated with 800 mg/kg MEPO, group B showed an insignificant (p = 0.284) increase in body weight when the pre-administration and post-administration body weights were compared (Table I).

### Reproductive hormones

FSH levels were increased across treated groups when compared to the control group but were only significant in the group treated with a higher dose of MEPO. There was no significant difference in the LH levels of the treated groups when compared to the control. There was an insignificant (p = 0.089 and 0.194) decrease in TT levels across all treated groups compared to the control (Table I).

### Epididymal sperm assessment

Table II shows the sperm count, motility, and morphology of adult Wistar rat following the administration of MEPO. The sperm count was significantly decreased (p = 0.006) in animals treated with a high dose of MEPO (800 mg/kg) when compared to the control group. The highest percentage of actively motile sperm was seen in group A (88%). The percentage of actively motile sperm was significantly reduced (p = 0.005 and 0.003, respectively) in group B (50%) and Group C (47%) compared to group A due to MEPO administration.

There was an increase in the percentage of sluggishly motile and non-motile sperm in groups B and C compared to control, but this was only significant (p = 0.002 and 0.023, respectively) for sluggish motility. There was no significant difference in the percentage of morphologically abnormal sperms and pus cells across all study groups (p = 0.434 and 0.422).

### Histopathological findings

#### Epididymal histology

**Figure 1:**
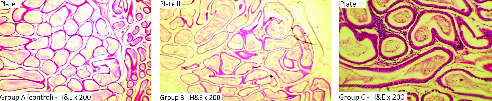
Histological section of the epididymis of rat. Plate I: Group A (control, only distilled water), Plate II: Group B (400 mg/kg of MEPO), Plate III: Group C (800 mg/kg of MEPO) (H&E × 200). MEPO: Methanolic extract of *Portulaca oleracea.*

Figure 1 shows plate's I-III. Plate I (group A) served as the control group and represents the histological section of the epididymis of rat administered only distilled water for 14 days. The photomicrograph shows a normal epididymis with characteristic stereociliated epithelium, spermatozoa in the lumen, basal cells, sperm cell, and loose connective tissue. Staining was done using H&E with photomicrography taken at × 200. Plate II (group B) shows the histological section of the epididymis of rat administered 400 mg/kg of MEPO for 14 days.

The section shows congested blood vessels, stereociliated epithelium, spermatozoa in a lumen, basal cells, and loose connective tissue. Staining was done using H&E with photomicrography taken at × 200. Plate III (group C) shows the histological section of the epididymis of rat administered 800 mg/kg of MEPO for 14 days. Histological sections of epididymis show congested blood vessels (arrow), stereociliated epithelium, spermatozoa in the lumen, basal cells, loose connective tissue. Staining was done using H&E with photomicrography taken at × 200.

#### Testicular histology

**Figure 2:**
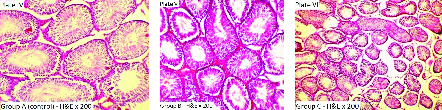
Histological section of the testis of rat. Plate I: Group A (control, only distilled water), Plate II: Group B (400 mg/kg of MEPO), Plate III: Group C (800 mg/kg of MEPO) (H&E × 200). MEPO: Methanolic extract of *Portulaca oleracea.*

Figure 2 shows plates IV-VI. Plate IV served as the control group (group A) - histological section of the testis of rat administered only distilled water for 14 days. The micrograph shows a normal testis. Staining was done using H&E with photomicrography taken at × 200. Plate V (group B) represents the histological section of rat testis administered 400 mg/kg of MEPO for 14 days.

The section shows tubules lined by spermatogenic series cells and containing numerous luminal spermatozoa. Staining was done using H&E with photomicrography taken at × 200. Plate VI (group C) shows the histological section of rat testis administered 800 mg/kg of MEPO for 14 days. The section shows tubules lined by spermatogenic series cells and containing numerous luminal spermatozoa. Staining was done using H&E with photomicrography taken at × 100.

**Table 1:** The effect of MEPO on the body weight, FSH, LH, and TT of adult Wistar rat


	**Group A**	**Group B**	**Group C**	**P-value**
Body weight (gr)*					
	**Pre-administration**	173.40 ± 4.91	157.20 ± 34.58	217.60 ± 5.89	0.006${}^{a}$ 0.284 0.041${}^{a}$
	**Post-administration**	200.00 ± 0.00*	200.00 ± 0.00	200.00 ± 0.00*	
Hormones**					
	**FSH (mIU/ml)**	0.41 ± 0.03	0.44 ± 0.01	0.50 ± 0.01*	- 0.396 0.020${}^{a}$
	**LH (mIU/ml)**	1.79 ± 0.10	2.11 ± 0.050	2.18 ± 0.44	- 0.428 0.359
	**TT (ng/ml)**	0.34 ± 0.01	0.18 ± 0.05	0.22 ± 0.07	- 0.089 0.194
Data presented as Mean ± SEM. *Analyzed using student dependent *t* test. **Analyzed using one-way ANOVA, followed by LSD. ${}^{a}$Significant at p < 0.05. MEPO: Methanolic extract of *Portulaca oleracea,* FSH: Follicle-stimulating hormone, LH: Luteinizing hormone, TT: Testosterone. Group A: Control (only distilled water), group B: Received 400 mg/kg of MEPO, group C: Received 800 mg/kg of MEPO					

**Table 2:** The effect of MEPO on sperm count, motility, and morphology of adult Wistar rat


**Sperm motility (%)**	**Group A**	**Group B**	**Group C**	**P-value**
**Actively motile**	87.67 ± 1.45	50.00 ± 5.77	46.67 ± 0.33	- 0.005* 0.003*
**Sluggishly motile**	10.00 ± 1.15	40.00 ± 5.77	26.67 ± 3.33	- 0.002* 0.023*
**Non-motile**	2.33 ± 0.33	10.00 ± 0.00	26.67 ± 12.01	0.113
**Sperm count (x10${}^{6}$)**	18.37 ± 2.65	12.43 ± 1.14	7.50 ± 1.39	- 0.064 0.006*
**Sperm morphology (%)**	83.33 ± 1.67	86.00 ± 1.00	81.67 ± 3.33	0.434
Data presented as Mean ± SEM. Data were analyzed using one-way ANOVA followed by post-hoc Fisher's LSD multiple comparisons. *Significant at p < 0.05. MEPO: Methanolic extract of *Portulaca oleracea. *Group A: Control (only distilled water), group B: Received 400 mg/kg of MEPO, group C: Received 800 mg/kg of MEPO				

## 4. Discussion

The present study evaluated the reproductive changes following MEPO administration in adult Wistar rats. Our findings showed that there was a significant increase in body weight in the control group whereas the extract administration caused a significant decrease in animals treated with a high dose of MEPO (800 mg/kg) (Table I). The phytochemical components of PO may play a role in the bodyweight reduction, although not reported in this article. One of the authors of this study had previously identified tannin as one of the major constituents of PO, which is associated with appetite reduction and may lead to fat store depletion (7). Our study is not too different from previous findings that reported no difference in the animal body weight after treatment with aqueous, methanolic, and ethanolic extracts of PO (20). However, it does not agree with another finding that showed a significant increase in the animal body weight following the administration of hydroalcoholic extract of PO (21).

There was no significant difference between the serum hormones of the treatment and control study groups observed in this study except for the FSH, which was seen to be significantly increased at a higher dose (800 mg/kg) of MEPO (Table I). The significant increase in FSH in group C (800 mg/kg) could be due to negative feedback induced on the hypothalamus and the pituitary gland by the low TT level, as high TT is required for spermatogenesis (22). FSH stimulates the Sertoli cells to produce androgen-binding receptors to support spermatogenesis and sperm cell maturation (22). The insignificant decrease in TT levels reported in this study suggests that the extract inhibits the mechanism intervening in the process of hormone synthesis in the Leydig cells, since it has been reported that the amount of TT produced by existing Leydig cells is under the control of LH, thereby explaining the similarity in our observations in LH and TT (22). LH stimulates secretion of sex steroids from the gonads and binds to receptors on Leydig cells, stimulating synthesis and secretion of TT (22); thus, the insignificant increase in LH as observed suggests that more LH stimulation may be needed to stimulate the Leydig cells for TT production (22). Our findings were the same as some previous studies, as they reported a significant increase in FSH levels following the administration of ethanolic extract of PO (20, 21). Howbeit, our findings disagrees with an earlier report of a significant decrease in TT level following the administration of PO extracts (13).

There were varying observations in the epididymal sperm analysis reported in this study (Table II). While there was no significant difference in the percentage of morphologically abnormal sperms and pus cells across all study groups (data not shown), there was a significant decrease in the sperm count of the animals treated with 800 mg/kg of MEPO. More so, the percentage of actively motile sperm was significantly reduced in a dose-dependent fashion (p = 0.005 and 0.003, respectively) in MEPO-treated groups (50% and 47%, respectively) when compared to the control group (Table II); howbeit, the non-motile sperm count was seen to be same across all the experimental groups.

The anti-spermatogenic and mobility-diminishing effect observed in MEPO-treated groups may be associated with the decrease in their serum TT, though insignificant (p = 0.089 and 0.194). The mechanism for the reduction in motility of sperm and sperm count induced by MEPO is not clearly understood and may need further research to be fully explored. Our findings agree with the reports by some studies which also showed a significant reduction in sperm motility, count, and viability as a result of the administration of crude PO extracts (1, 13). However, a recent study observed a significant increase in sperm count in their study after the administration of both lipophilic and hydrophilic extracts of PO even in the presence of reduced serum TT (23). This differing outcome remains unclear.

The histological sections of the epididymis (Figure 1) observed in the present study presented a normal epididymis with stereociliated epithelium, spermatozoa in the lumen, basal cells, sperm cells, and loose connective tissue - a completely normal histoarchitecture. However, a dose-dependent vascular congestion was noted in the MEPO-treated groups. This will be the first study to the best of our knowledge documenting the effect of MEPO on the epididymis of adult Wistar rats. The epididymal congestion may have contributed to the reduced sperm count and motile sperms observed in this study due to the role it plays in tissue oxidative stress and cellular damage (11). The histological findings of testicular tissue (Figure 2) showed tubules lined by spermatogenic series cells and containing numerous luminal spermatozoa. We observed no abnormalities in the histoarchitecture of the testicular tissues. This report contradicts an earlier study which documented that the crude extracts of PO caused acellular seminiferous tubules as well as Leydig cell hyperplasia (13). They also observed fibrosis of the stroma after the ingestion of crude extracts of PO. This varying outcome may be as a result of the long duration of PO administration.

The effects observed in our study may have been affected by the study duration, the route and dosage of administration of PO extract, thus the reasons for the differences in the tissue-based effect observed. More so, some of the available studies describing the effects of PO extract on the male reproductive function made use of isolates and singular fractions from PO which could lead to a different research outcome.

## 5. Conclusion

This study has shown some evidence on the spermatotoxic effects of MEPO and its sperm maturation arrest potential by vascular congestion in the epididymis, notwithstanding its non-significant effect on the male reproductive hormones and the testis. A chronic study should be conducted to validate the claims made in the present study and to elucidate the mechanism of action for the spermatotoxic effects following the administration of MEPO.

##  Conflict of Interest

There is no conflict of interest to declare.
